# The relationship between digital resource allocation and digital literacy of kindergarten teachers: the chain mediating effect of self-efficacy and learning motivation

**DOI:** 10.3389/fpsyg.2025.1639007

**Published:** 2025-09-17

**Authors:** Fei He, Liying Wen

**Affiliations:** ^1^Inner Mongolia Autonomous Region, School of Educational Science, Inner Mongolia University for Nationalities, Tongliao, China; ^2^Research Center for Student Bullying Prevention and Control, Inner Mongolia Autonomous Region, Tongliao, China

**Keywords:** digital literacy, digital resource allocation, self-efficacy, learning motivation, kindergarten teachers

## Abstract

**Introduction:**

Research on digital literacy has become deeply integrated into the field of teacher education, significantly influencing educational practices, including kindergarten education. For kindergarten teachers, possessing adequate digital literacy is essential for effectively conducting teaching activities and improving overall work efficiency. However, existing studies on digital literacy primarily focus on assessing individual teacher competency levels or conducting macro-level analyses of national policies.

**Methods:**

This study examines the correlation mechanism between digital resource allocation and the digital literacy of kindergarten teachers. The research sample includes 317 kindergarten teachers from southeastern China. Data were analyzed using SPSS 29.0 and Mplus 8.0.

**Results:**

The results reveal that digital resource allocation is significantly and positively correlated with kindergarten teachers’ digital literacy. Moreover, both self-efficacy and learning motivation serve as mediating factors in this relationship. Notably, self-efficacy and learning motivation also form a chain mediation effect between digital resource allocation and digital literacy. Additionally, the direct effects of these factors on digital literacy, while present, are not significant.

**Discussion:**

These findings underscore the importance of digital resource allocation, self-efficacy, and learning motivation in enhancing digital literacy among kindergarten teachers. The study provides valuable insights for developing strategies to support and improve digital literacy in kindergarten teachers.

## 1 Introduction

Digital literacy has long been considered one of the basic abilities of teachers. It is defined as an individual’s ability to discover and evaluate information, effectively use this information to create new content, and share and exchange new works through digital technologies (Reddy et al., 2020). In other words, digital literacy involves the confident and critical use of a full range of digital technologies for information, communication and basic problem-solving in all aspects of life. It is underpinned by basic skills in ICT`: the use of computers to retrieve, assess, store, produce, present and exchange information, and to communicate and participate in collaborative networks via the Internet (United Nations Educational, Scientific and Cultural Organization, n.d.). Kindergarten teachers with high digital literacy are more willing to apply digital tools in teaching, which improves their work efficiency (Reddy et al., 2021; Lim, 2023). Some studies state that kindergarten teachers can conduct teaching activities more vividly by using digital technologies (Zhao and Li, 2015; Wolak and Kim, 2023). For example, they use language, images, sound, and digital tools and technologies to create content in various new forms (Zhao and Li, 2015). Conversely, studies have also found that if kindergarten teachers are not proficient in using digital technologies, their workload can increase, ultimately reducing their efficiency (Wolak and Kim, 2023). Therefore, exploring the associative mechanisms between digital resource allocation and kindergarten teachers’ digital literacy and developing effective improvement strategies can help enhance their digital literacy levels. Previous research from different perspectives—Previous studies on teachers (Zhang, 2023), education in kindergarten (You and Yu, 2025), as well as national-level education system (Munawar et al., 2021) has shown that positive attitudes toward digital technologies (Hobbs and Tuzel, 2017), digital training, and digital policies (Munawar et al., 2021) may affect the development of digital literacy of teachers. Previous studies have mainly examined digital literacy from the perspectives of teacher characteristics, such as the digital competence framework of the European Commission and the policy guidelines of the United States (Sánchez-Cruzado et al., 2021; Martindale et al., 2024; Redecker and Punie, 2017), often overlooking the associative mechanisms between influencing factors and digital literacy. In particular, the connections between material conditions, individual psychology, and digital literacy have been underexplored. Considering that material conditions (e.g., resource allocation) and psychological characteristics (e.g., self-efficacy, motivation) are important prerequisites for digital literacy development, this study draws on self-efficacy theory and learning motivation theory. It examines how digital resource allocation predicts kindergarten teachers’ digital literacy and explores the associative mechanisms between digital resource allocation and kindergarten teachers’ digital literacy by means of self-efficacy and learning motivation factors.

There are ongoing debates in academic circles regarding the definition of digital literacy. Gilster and Gilster (1997), who first proposed the concept, defined digital literacy as the ability to understand and use information through computers. They viewed digital literacy as a broad ability to comprehend and utilize digital information in the digital age. Martin and Grudziecki (2006) further defined digital literacy as the awareness, attitudes, and abilities of individuals to identify, acquire, manage, integrate, evaluate, analyze, and synthesize digital resources using digital tools in specific life contexts. Compared to Gilster (1997), Martin and Grudziecki (2006) offered a more detailed definition, emphasizing specific digital skills and the importance of social contexts. Additionally, the European Union considers digital literacy a new concept derived from other literacies, encompassing information literacy, media literacy, network literacy, and computer literacy (Ala-Mutka, 2011). Furthermore, Ala-Mutka (2011) provides a foundational conceptual framework for digital competence, distinguishing three interrelated dimensions: (a) instrumental knowledge and skills related to tools and media; (b) advanced abilities in communication, collaboration, information management, learning, and problem-solving; and (c) strategic attitudes supporting critical, creative, responsible, and autonomous engagement with digital technologies. Building on this, UNESCO redefined digital literacy by examining specific digital capabilities, social contexts, and related literacies. According to UNESCO, digital literacy is the ability of individuals to access, manage, understand, integrate, communicate, evaluate, and create information using digital technologies in the context of employment, decent work, and entrepreneurship (Law et al., 2018). This definition includes computer operation skills, information and communication technology knowledge, information literacy, and media literacy, clearly outlining specific capabilities, identifying relevant social contexts, and clarifying the relationship between digital literacy and related literacies (Zhang and Sheng, 2019), aligning with international perspectives. Based on these definitions, and considering the integrated nature of care and education in Chinese kindergartens (Pulimeno et al., 2020), along with the multiple roles, complex tasks, and high work pressure faced by kindergarten teachers (Yue and Ji, 2017), this study defines kindergarten teachers’ digital literacy as the ability to use digital technologies to access digital resources and enhance their professional capabilities. This includes comprehensive abilities such as critical innovation, teaching and learning application, and collaboration (Nguyen and Habók, 2024), as well as digital moral literacy, capability-building literacy, and digital collaborative literacy.

### 1.1 Digital resource allocation and digital literacy of kindergarten teachers

Digital resource allocation refers to the digital facilities and equipment (including software, hardware, and training) provided by countries or governments to schools, aimed at serving teachers and students and enhancing teachers’ digital skill levels (Edumadze and Owusu, 2013). Digital resource allocations are based on individuals’ digital literacy levels (Hinrichsen and Coombs, 2014). Research perspectives on digital resource allocation and digital literacy vary. Mikelić Preradović et al. (2017) found that countries such as Croatia lack digital technology support in early childhood education, with teachers also lacking relevant digital knowledge. These teachers primarily rely on traditional teaching media, which may, to some extent, weaken their digital literacy. In contrast, the U. S. government has equipped kindergartens with adequate digital tools, enabling teachers to integrate technologies such as sensors, computers, and wireless communication into teaching, thereby helping to improve their digital literacy (Organization for Economic Co-operation and Development, 2023). Similarly, the Finnish government has made substantial investments in digital infrastructure, allowing teachers to apply digital tools in the classroom, potentially enhancing their digital literacy (Vartiainen et al., 2019). In the context of China, studies have shown that kindergartens in Hong Kong are equipped with relatively comprehensive digital infrastructure, enabling teachers to integrate technologies such as augmented reality into curriculum activities (Huang et al., 2015). However, research has also revealed imbalances in digital resource allocation between developed and underdeveloped regions in mainland China, which are reflected in corresponding disparities in teachers’ digital literacy levels (Fan et al., 2024).

Studies indicate that teachers working in environments with richer digital resources generally possess higher digital literacy (Nguyen and Habók, 2024). Abundant digital resources can support teaching practices, enhancing teachers’ enthusiasm and effectiveness in applying technology in kindergarten classrooms (Wolak and Kim, 2023). Further, existing research confirms that digital resource allocation has a significant positive impact on teachers’ digital literacy (Eryansyah et al., 2020). For example, the computer center at Coast Angle University supports the development of teaching materials by offering rich digital resources and computer-based training, effectively promoting digital integration in teaching (Edumadze and Owusu, 2013). In other words, when teachers have access to abundant digital resources at work, they are more likely to use digital devices in daily teaching, which can improve their digital literacy (Quaicoe and Pata, 2020). Therefore, Saikkonen and Kaarakainen (2021) argue that without improving digital infrastructure and allocating resources effectively, schools will lack the basic conditions needed to support digital teaching. Specifically for kindergarten teachers, access to more digital resources can help enhance their digital literacy. Thereby, this study proposes the first hypothesis (H1): digital resource allocation is positively correlated with kindergarten teachers’ digital literacy level. That is, kindergartens with more comprehensive digital resource allocation typically have teachers with higher digital literacy levels.

### 1.2 Digital resource allocation, self-efficacy, and digital literacy of kindergarten teachers

Self-efficacy refers to an individual’s belief in their capability to organize and execute actions required to manage prospective situations (Bandura, 1977, 2006). It is typically understood as a domain-general psychological construct that shapes confidence across different types of tasks. In the present study, we focus specifically on teachers’ *digital self-efficacy*, that is, their perceived confidence in using digital technology resources effectively in their teaching practice. This operational definition emphasizes teachers’ subjective assessments of their ability to adopt and integrate digital tools into kindergarten teaching activities. Throughout this manuscript, the term self-efficacy refers to this digital technology–related self-efficacy. When individuals receive sufficient support through digital resource allocation, their self-efficacy in using digital tools is significantly enhanced (Sun and Yan, 2025). Rich digital resources help individuals accumulate experience with digital technologies, thereby improving their self-efficacy (Chao et al., 2016). In other words, individuals with greater access to digital resources typically exhibit stronger self-efficacy. Self-efficacy might play a mediating role between digital resource allocation and digital literacy. Research shows that a lack of essential digital resources in schools may significantly reduce teachers’ self-efficacy (Pajares, 1997). Moreover, studies have confirmed a significant correlation between digital resource allocation and self-efficacy (Vancouver et al., 2008; Beck and Schmidt, 2015). Regarding the relationship between self-efficacy and digital literacy, individuals with high self-efficacy often possess higher levels of digital literacy. Existing research has highlighted a strong association between self-efficacy and digital literacy (Bandura, 2006; Lim, 2023; Sun and Shi, 2024). Specifically, individuals with high self-efficacy are more capable of improving their digital literacy skills (Lilian, 2022) and tend to exhibit stronger online learning abilities (Prior et al., 2016; Rohatgi et al., 2016). According to self-efficacy theory, individual behavioral performance is directly influenced by their self-efficacy level (Bandura, 1977). In the educational context, teachers with high self-efficacy are more inclined to use digital technologies to acquire knowledge, thereby enhancing their digital literacy (Prior et al., 2016). Such teachers are also more likely to proactively adopt digital tools and master relevant knowledge (Awofala et al., 2019). Specifically for kindergarten teachers, having high self-efficacy during the teaching process can contribute to improved digital literacy. Therefore, this study proposes the second hypothesis (H2): self-efficacy plays a mediating role between digital resource allocation and kindergarten teachers’ digital literacy. That is, the more abundant the digital resources in kindergartens, the higher the teachers’ self-efficacy, which may in turn enhance their digital literacy.

### 1.3 Digital resource allocation, learning motivation, and digital literacy of kindergarten teachers

Learning motivation generally refers to the internal processes that initiate, guide, and sustain individuals’ efforts to acquire new knowledge or skills (Gopalan et al., 2017). While prior research often emphasizes students’ learning motivation, it is equally important to examine teachers’ learning motivation, as teachers must continually learn and adapt in their professional roles. In this study, *learning motivation* refers to kindergarten teachers’ willingness and drive to learn how to apply digital technology resources in their work. This includes both the intrinsic desire to explore new teaching methods using digital tools and the extrinsic motivation stimulated by resource availability and institutional expectations. Furthermore, motivation can be divided into intrinsic motivation and extrinsic motivation. Intrinsic motivation leads to an activity carried out merely for one’s own satisfaction, interest or challenge without any expectation of rewards. Therefore, in education, optimal perseverance and a positive attitude are needed to maintain intrinsic motivation. Specifically, intrinsic motivation affects the effectiveness of an individual’s learning of digital technology, which directly influences an individual’s digital literacy. Whereas extrinsic motivation is driven by external activities, such as rewards, pressure, and punishment. Therefore, motivation can be cultivated. Specifically in education, rich digital resource allocation is an extrinsic incentive, beneficial to an individual’s extrinsic motivation, which in turn affects an individual’s digital literacy. According to Gopalan et al. (2017), digital learning environments that meet psychological and instructional needs promote teachers’ learning motivation. Rich digital resources serve this role.

Thus, high levels of digital resource allocation can help boost teachers’ learning motivation (Lin et al., 2017). In educational environments with abundant digital resources, teachers can more effectively integrate and apply these tools in their teaching, which in turn stimulates their motivation to learn. Existing research has shown that digital resource allocation can significantly improve teachers’ learning motivation and interest in teaching (Chao et al., 2016; Lin et al., 2017). Rich digital resources offer teachers greater flexibility, enabling them to access teaching materials and solve problems through digital tools, thereby further enhancing their motivation to learn (Lee et al., 2015; Chao et al., 2016). Regarding the relationship between learning motivation and digital literacy, individuals with stronger learning motivation generally exhibit higher levels of digital literacy. Gopalan et al. (2017) learning theory highlights motivation as a key factor driving individuals to take action to achieve goals or fulfil needs. The development of digital literacy is closely linked to motivational guidance. Learning motivation but also encourages more efficient and conscious use of digital tools, thereby improving digital literacy (Lilian, 2022). That is, individuals with higher learning motivation are more inclined to use digital resources to acquire knowledge, thus enhancing their digital literacy. Additionally, research has found that students with high learning motivation more actively engage with internet technologies (Lin et al., 2017). Following Gopalan’s learning motivation framework, we posit that digital resource allocation enhances learning motivation, which mediates the relationship with digital literacy (Gopalan et al., 2017). Therefore, this study proposes the third hypothesis (H3): learning motivation plays a mediating role between digital resource allocation and kindergarten teachers’ digital literacy. This means, the more abundant the digital resources in a kindergarten, the higher the teachers’ learning motivation, which may further promote the improvement of their digital literacy.

### 1.4 Relationship between self-efficacy and learning motivation

Self-efficacy and learning motivation may play a chain-mediated role between digital resource allocation and digital literacy. Research shows that individuals with higher self-efficacy tend to exhibit stronger learning motivation (Pajares, 1997). Self-efficacy directly influences learning motivation by affecting task selection tendencies—individuals are more likely to engage in tasks they feel confident in and avoid those they doubt their ability to complete (Pajares, 1997). Digital resource allocation is an environmental factor that enhances personal belief in digital capability (self-efficacy), as per Bandura (1977, 2006). Therefore, this study hypothesizes a positive correlation between self-efficacy and learning motivation. Meanwhile, digital resource allocation can positively predict teachers’ digital literacy levels by enhancing both self-efficacy and learning motivation. Existing research suggests that when institutions are equipped with sufficient digital resources, teachers are better able to use operational tools, office software, communication platforms, and network technologies, which increases their confidence and intrinsic motivation in using digital technologies, thereby effectively improving their digital literacy (Saikkonen and Kaarakainen, 2021). Furthermore, teachers with high self-efficacy are more willing to invest effort in completing teaching tasks, which also contributes to improving their digital literacy (Lilian, 2022). Based on this, the study proposes the fourth hypothesis (H4): self-efficacy and learning motivation play a chain-mediated role between digital resource allocation and kindergarten teachers’ digital literacy. In other words, when digital resource allocation in kindergartens is more comprehensive, teachers have more opportunities for learning, which enhances their self-efficacy; this, in turn, stimulates stronger learning motivation, ultimately promoting their digital literacy.

## 2 Materials and methods

### 2.1 Research procedure

This study investigates the mediating roles of self-efficacy and learning motivation in the relationship between digital resource allocation and kindergarten teachers’ digital literacy. Using random sampling, a questionnaire survey was conducted among kindergarten teachers in southeastern China. Prior to the survey, researchers clearly explained the study’s purpose, and all participants provided informed consent, with the freedom to withdraw at any time. The questionnaire excluded sensitive information such as names or kindergarten affiliations to safeguard participants’ privacy. After organizing and screening the responses, invalid questionnaires were removed, resulting in 317 valid entries. The study strictly followed ethical guidelines and ensured the confidentiality of all personal information. As a gesture of appreciation, participants received electronic red packets valued between 2 and 5 yuan upon completing the survey.

### 2.2 Participants

The southeastern region of China has a relatively high level of economic development and has invested significantly in digital education, which reflects a typical environment for “digital literacy” research. Therefore, this study used random sampling to recruit kindergarten teachers from the southeastern region of China to participate in a questionnaire survey. All participants took part voluntarily and were informed that they could withdraw at any stage of the investigation. A total of 318 questionnaires were collected, 317 valid questionnaires were retained and used for subsequent data analysis, resulting in an effective rate of 99.69%. Ethical approval for this study was obtained from the Ethics Committee of the Medical and Life Sciences at Inner Mongolia Minzu University (approval number: NMD-RT-1025-08-01). Table 1 presents the demographic information of the respondents, including teachers’ sex, age, teaching experience, income level, educational background, region, and kindergarten type.

**Table 1:** Demographic information of kindergarten teacher sample (*N* = 317).

Demographic characteristic	Category	*N*	%
Sex	1. Male	48	15.1%
	2. Female	269	84.9%
Age (years)	1. ≤ 20	21	6.60%
	2. 21–30	215	67.80%
	3. 31–40	51	16.10%
	4. 41 and above	30	9.50%
Kindergarten type	1. Public kindergarten	213	67.20%
	2. Private kindergarten	104	32.80%
Region of kindergarten	1. Urban	186	58.70%
	2. Rural	131	41.30%
Educational background	1. High school and vocational school	21	6.60%
	2. Junior college	110	34.70%
	3. Bachelor’s degree	173	54.60%
	4. Master’s degree	13	4.10%
Teaching experience	1. ≤1 year	64	20.20%
	2.2-4 years	131	41.30%
	3.5–10 years	94	29.70%
	4. ≥ 11 years	28	8.80%
Income	1. < 2000 yuan	43	13.60%
	2.2000–4,000 yuan	160	50.50%
	3.4000–6,000 yuan	80	25.20%
	4. > 6,000 yuan	34	10.70%

1 USD = 7.1907 yuan.

### 2.3 Measures

#### 2.3.1 Digital literacy questionnaire

The kindergarten teacher digital literacy questionnaire used in this study is based on 22 capabilities from the digital literacy framework for English educators and was adapted from a digital literacy survey questionnaire for primary and secondary school teachers (Peng and Zhu, 2024). To ensure the accuracy and reliability of the scale, two researchers translated and back-translated the 22 capabilities from. Based on this, we localized the questionnaire by incorporating the actual context of kindergarten education in China. Four experts were then invited to assess the content reliability of the questionnaire, and a small-scale pilot test was conducted, and 20 kindergarten teachers were included in the test. Based on the pilot results, 2 items were deleted, and 12 items were retained, forming the final questionnaire. The questionnaire evaluates five core dimensions of kindergarten teachers’ digital literacy: digital knowledge (e.g., “I am familiar with the knowledge of digital technology tools commonly used in kindergartens.”), digital awareness (e.g., “I know how to use digital technology to obtain information resources in daily educational activities.”), information processing ability (e.g., “I can effectively analyze online information while using digital technology to obtain resources in daily teaching.”), technological operation ability (e.g., “I can proficiently use basic office software.”), and communication and sharing ability (e.g., “I can share teaching experiences of applying digital technology in kindergartens through platforms such as Xiaohongshu, Weibo, and TikTok.”). All items in the questionnaire use a five-point Likert scale, ranging from 1 (strongly disagree) to 5 (strongly agree), with higher scores in each dimension indicating a higher level of digital literacy among kindergarten teachers. In this study, the Cronbach’s α coefficient of the scale was 0.942, with Cronbach’s α coefficients for each dimension ranging from 0.807 to 0.865, indicating good internal consistency. Additionally, the KMO value was 0.950, *χ*^2^/df = 1.403, RMSEA = 0.036, SRMR = 0.018, CFI = 0.993, and TLI = 0.990, indicating that the measurement tool has good structural validity and is suitable for assessing kindergarten teachers’ digital literacy.

#### 2.3.2 Digital resource allocation questionnaire

The Digital Resource Allocation Questionnaire is based on the framework of the “Compulsory Education Digital Resource Standards” and adapted from teacher digital educational resource-relate questionnaire (Chen et al., 2022). To ensure the scientific validity and applicability of the questionnaire, three experts were invited to assess content validity, and a small-scale pilot test was conducted, and 20 kindergarten teachers were included in the test. The final questionnaire contains four items (e.g., “Our kindergarten is equipped with relatively rich digital devices.”), using a five-point Likert scale ranging from 1 (strongly disagree) to 5 (strongly agree). In this study, the Cronbach’s α coefficient was 0.85, indicating good internal consistency. Additionally, the KMO value was 0.822, *χ*^2^/df = 0.411, RMSEA = 0.000, SRMR = 0.006, CFI = 1.000, and TLI = 1.007. These results suggest that the measurement tool has good structural validity and is suitable for assessing digital resource allocation in kindergarten teachers.

#### 2.3.3 Self-efficacy scale

The Self-Efficacy Scale was adapted from the New General Self-Efficacy Scale developed by Chen et al. (2001). To ensure the scale’s accuracy and reliability, two researchers translated and back-translated it. Subsequently, 3 experts were invited to evaluate the content validity of the scale, and a small-scale pilot test was conducted that included 20 kindergarten teachers. The test assessed the scale’s operability and applicability. The scale consists of eight items, four of which showed relatively high consistency with this research, and were selected. (e.g., “When facing difficult tasks, I am certain that I will accomplish them.”), each scored on a five-point Likert scale ranging from 1 (strongly disagree) to 5 (strongly agree), with higher total scores indicating higher levels of individual self-efficacy. This scale’s Cronbach’s α coefficient was 0.876, indicating good internal consistency. Additionally, the KMO value was 0.834, *χ*^2^/df = 1.147, RMSEA = 0.022, SRMR = 0.008, CFI = 1.000, and TLI = 0.999. These indicators suggest that the scale has good structural validity and is appropriate for measuring self-efficacy among kindergarten teachers in this study.

#### 2.3.4 Learning motivation scale

This scale was adapted from the Student Motivation Scale (Martin, 2003) to assess an individual’s level of intrinsic learning motivation in a specific context. To ensure the scale’s accuracy and reliability, two researchers translated and back-translated it. Moreover, three experts in the relevant field were invited to evaluate the content validity of the scale, and a small-scale pre-test was carried out to examine its operability and applicability. The scale includes 40 items, of which 3 items having relatively high consistency with this research were selected. Such as: “If I try hard, I believe I can learn digital technology well.”All items were scored using a five-point Likert scale, ranging from 1 (strongly disagree) to 5 (strongly agree), with higher total scores indicating stronger individual learning motivation. This scale’s Cronbach’s α coefficient was 0.842, indicating good internal consistency. Additionally, the KMO value was 0.727, *χ*^2^/df = 0.000, RMSEA = 0.000, SRMR = 000, CFI = 1.000, and TLI = 1.000. Since this is a saturated model, the test value is either 0 or 1.

### 2.4 Control variables

In this study, teachers’ sex, age, kindergarten type, and seniority were included as control variables, as these demographic characteristics may indirectly influence kindergarten teachers’ digital literacy levels.

### 2.5 Statistical analysis

This study employed SPSS 29.0 and Mplus 8.0 to perform statistical analyses on the collected data. Specifically, SPSS was used for testing common method bias, conducting descriptive statistics, correlation analysis, and assessing scale reliability. Mplus 8.0 was used for validity testing, model fit evaluation, and mediation effect analysis. Upon confirming a good model fit, Mplus was further applied to examine the chain mediation effects and underlying mechanism of self-efficacy and learning motivation between digital resource allocation and kindergarten teachers’ digital literacy. To assess the significance of total, direct, and mediating effects in the mediation model, the study used the Bootstrap method with 5,000 resamples and constructed a 95% confidence interval. An effect is deemed statistically significant if the confidence interval does not include 0 (Wen and Ye, 2014).

## 3 Research results

### 3.1 Common method bias test and multicollinearity diagnostic test

This study employed self-reported measures, which may be subject to common method bias. To mitigate its potential impact, the Harman single-factor test was conducted (Podsakoff et al., 2003). The unrotated factor analysis revealed five factors with eigenvalues greater than 1, and the first factor accounted for 38.6% of the variance—below the critical threshold of 40% (Podsakoff et al., 2003). This indicates that common method bias was not a significant concern. Additionally, SPSS was used to assess multicollinearity among variables. The variance inflation factors were 2.978, 4.411, and 3.562, all below the threshold of 5; and the tolerance values were 0.336, 0.227, and 0.281, all exceeding 0.1. These results confirm the absence of serious multicollinearity. In sum, the data used in this study demonstrate strong reliability and stability (Hair et al., 2019).

### 3.2 Descriptive statistical analysis

Table 2 presents the correlation coefficients between the independent and dependent variable dimensions. All five subdimensions of digital literacy and digital resource allocation indicate significant positive correlation with each other as well as with self-efficacy, and learning motivation.

**Table 2:** Correlation analysis of digital literacy among kindergarten teachers and its influencing factors.

Variables	M ± SD	1	2	3	4	5	6	7	8	9	10	11
1. Digital literacy	3.97 ± 0.81	1										
2. Digital knowledge	3.85 ± 0.94	0.881^***^	1									
3. Digital awareness	4.05 ± 0.87	0.922^***^	0.757^***^	1								
4. Information processing capability	3.94 ± 0.92	0.851^***^	0.706^***^	0.776^***^	1							
5. Technical operational ability	4.04 ± 0.97	0.854^***^	0.667^***^	0.744^***^	0.617^***^	1						
6. Communication and sharing capabilities	3.99 ± 0.98	0.822^***^	0.601^***^	0.686^***^	0.626^***^	0.715^***^	1					
7. Digital resource allocation	3.95 ± 0.85	0.795^***^	0.673^***^	0.721^***^	0.698^***^	0.700^***^	0.665^***^	1				
8. self-efficacy	3.96 ± 0.87	0.833^***^	0.702^***^	0.757^***^	0.713^***^	0.739^***^	0.713^***^	0.803^***^	1			
9. Learning motivation	3.94 ± 0.88	0.773^***^	0.667^***^	0.685^***^	0.655^***^	0.662^***^	0.694^***^	0.749^***^	0.839^***^	1		
10. Sex	1.85 ± 0.36	0.069	0.067	0.053	0.079	0.012	0.088	−0.004	0.026	0.005	1	
11. Age	2.28 ± 0.73	−0.135^*^	−0.091	−0.126^*^	−0.065	−0.143^*^	−0.170^**^	−0.159^**^	−0.175^**^	−0.145^**^	−0.126^*^	1

^*^p < 0.05, ^**^p < 0.01, and ^***^p < 0.001.

### 3.3 Model verification

This study employed Mplus 8.0 to perform model fit analysis, yielding the following fit indices: χ^2^/df = 3.480, RMSEA = 0.088, SRMR = 0.038, CFI = 0.943, and TLI = 0.932. Although the RMSEA is greater than 0.08, it is still within the acceptable range. Meanwhile, other indicators are good.

### 3.4 Mediation model

**Figure 1:**
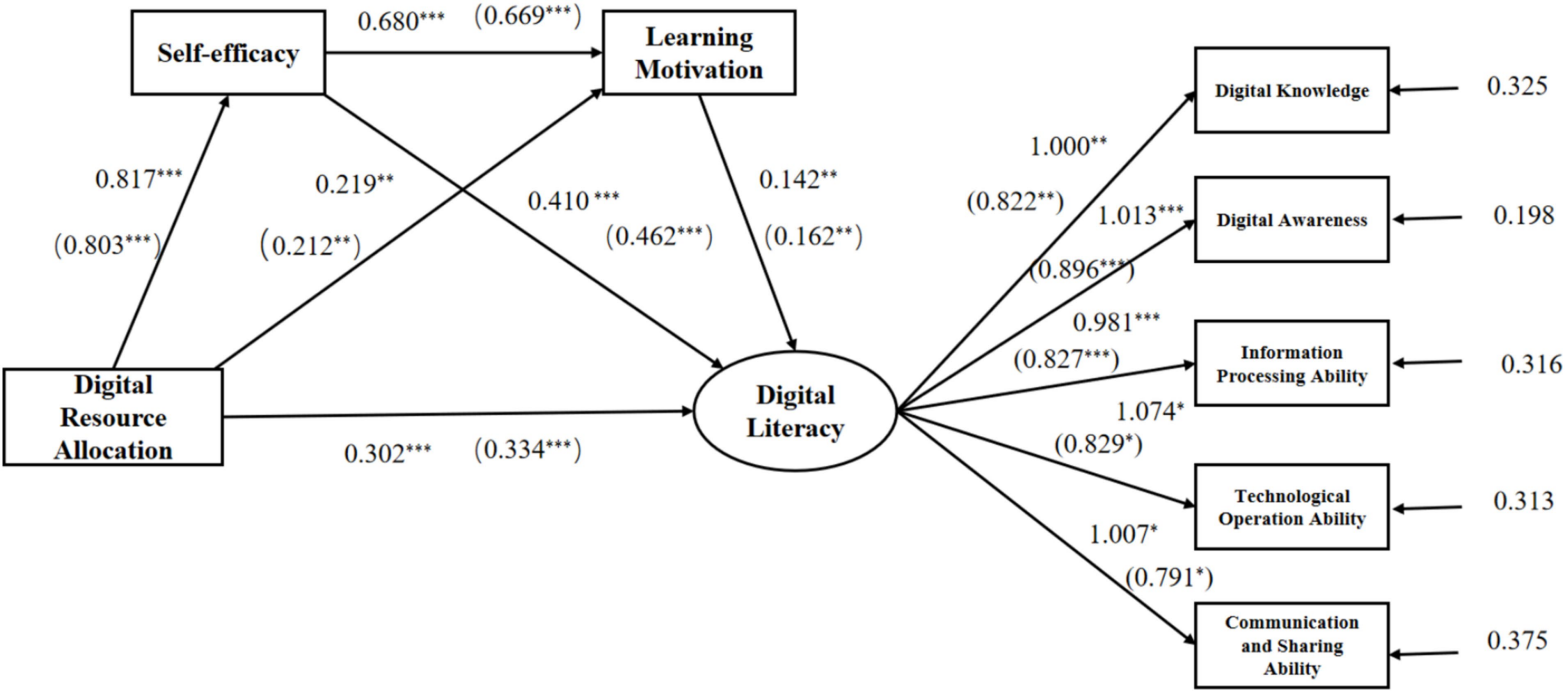
Relationship between digital resource allocation and digital literacy: The mediating role of self-efficacy and learning motivation. *p < 0.05, **p < 0.01, and ***p < 0.001. The coefficients in parentheses are standardized coefficients. The items outside the parentheses are non-standardized coefficients. Source: Authors’ own creation.

To verify the research hypotheses, this study employed Mplus 8.0 to examine the mediation effects. The model included digital resource allocation, self-efficacy, learning motivation, and kindergarten teachers’ digital literacy as outcome variables, with digital resource allocation as the predictor variable and self-efficacy and learning motivation as mediating variables. The aim was to explore the potential mediating relationships between digital resource allocation and the outcome variables. Figure 1 and Table 3 present the analysis results. Mplus 8.0 was also used to assess the significance of total, direct, and indirect effects, and the Bootstrap method (with 5,000 iterations and a confidence interval of 95%) was employed to test and construct the chain mediation model.

**Table 3:** Mediating role of self-efficacy and learning motivation.

Path	Std. Est	S. E	Est./S. E	Boot LLCI	Boot ULCI	Effect ratio
Digital resource allocation → Self-efficacy → Digital literacy	0.335	0.051	6.604	0.236	0.434	44.85%
Digital resource allocation→ Learning motivation → Digital literacy	0.031	0.015	2.123	0.002	0.06	4.15%
Digital resource allocation → Self-efficacy → Learning motivation → Digital literacy	0.079	0.027	2.877	0.025	0.132	10.58%
Direct effect	0.302	0.058	5.795	0.2	0.405	40.43%
Total indirect effect	0.445	0.047	9.547	0.353	0.536	59.57%
Total effect	0.747	0.044	16.869	0.66	0.833	100.00%

The structural equation model results (Figure 1) indicated a significant positive relationship between digital resource allocation and kindergarten teachers’ digital literacy, supporting H1. This suggests that greater digital resource allocation in kindergartens is associated with higher levels of teachers’ digital literacy. Furthermore, both self-efficacy and learning motivation were significantly positively associated with teachers’ digital literacy, indicating that teachers with higher self-efficacy or stronger learning motivation tend to exhibit higher levels of digital literacy. Additional analysis revealed significant positive correlations between digital resource allocation and self-efficacy, digital resource allocation and learning motivation, and self-efficacy and learning motivation. This indicates that increased digital resource allocation or higher self-efficacy enhances teachers’ learning motivation, which may subsequently contribute to improved digital literacy.

The indirect paths and corresponding path coefficients are summarized in Table 3. The results show that self-efficacy plays a significant mediating role between digital resource allocation and digital literacy, supporting H2. This suggests that teachers with greater access to digital resources exhibit higher self-efficacy in using digital tools for teaching, which enhances their digital literacy levels.

Learning motivation plays a mediating role in the relationship between digital resource allocation and digital literacy, supporting H3. This suggests that teachers with greater access to digital resources exhibit stronger motivation to use digital technologies in teaching, improving their digital literacy levels.

Self-efficacy and learning motivation jointly play a chain mediating role between digital resource allocation and digital literacy, supporting H4. In other words, when kindergartens are equipped with richer digital resources, teachers develop higher self-efficacy and learning motivation in using digital technologies, further enhancing their digital literacy.

The chain mediating effect of self-efficacy and learning motivation between digital resource allocation and digital literacy is significantly positively correlated with kindergarten teachers’ digital literacy. In conclusion, the configuration of digital resources in kindergartens, as a key aspect of the digital environment, influences teachers’ digital literacy in complex ways. This influence is not direct or linear but is transmitted and shaped through mediating variables such as self-efficacy and learning motivation. Additionally, learning motivation serves as another important mediator, further reinforcing this relationship. Certain aspects of digital resource allocation directly boost self-efficacy, which in turn enhances learning motivation, ultimately improving digital literacy through this chain of influence. This intricate mediating process highlights the complexity of interactions among these variables.

## 4 Discussion

This study explores the correlation mechanism between digital resource allocation and kindergarten teachers’ digital literacy and further examines the mediating roles of self-efficacy and learning motivation. The findings reveal significant correlations among digital resource allocation, teachers’ digital literacy, self-efficacy, and learning motivation. Digital resource allocation not only directly enhances teachers’ digital literacy but also indirectly improves it through a sequential mediation chain involving self-efficacy and learning motivation.

### 4.1 Digital resource allocation is significantly positively correlated with kindergarten teachers’ digital literacy level

Current research indicates a significant positive correlation between digital resource allocation and kindergarten teachers’ digital literacy. Kindergartens equipped with adequate hardware and software facilities enhance teachers’ ability to select and utilize digital tools in their teaching, thereby fostering the development of digital literacy (Voogt and McKenney, 2016). Moreover, studies suggest that a campus environment rich in digital resources provides teachers with opportunities to practice and apply digital technologies, further improving their digital literacy levels (Voogt and McKenney, 2016). Supporting this perspective, Eryansyah et al. (2020) highlight the vital role of on-campus digital resources in developing teachers’ digital skills (Eryansyah et al., 2020). Therefore, digital resource allocation is a key supporting factor in advancing kindergarten teachers’ digital literacy. Based on these findings, future educational practices may view digital resource allocation as an effective strategy for enhancing teachers’ digital literacy. However, some studies caution that merely improving digital infrastructure does not automatically result in higher digital literacy (Tomczyk, 2020). This may be closely linked to internal factors such as individual teachers’ knowledge, attitudes, cognition, beliefs, and digital skills. Therefore, alongside resource allocation, efforts must also focus on supporting teachers’ adaptation and capacity building to achieve a holistic improvement in digital literacy.

### 4.2 Mediating role of self-efficacy between digital resource allocation and kindergarten teachers’ digital literacy

The study further revealed that digital resource allocation significantly and positively influences kindergarten teachers’ digital literacy through self-efficacy, with a path coefficient of 44.85%. Results from the structural equation model analysis indicate a strong correlation between digital resource allocation and the development of teachers’ self-efficacy, aligning with previous research findings (Vancouver et al., 2008; Chao et al., 2016). According to self-efficacy theory, increased access to digital resources can stimulate individuals’ intrinsic motivation, encouraging more positive and proactive behavior (Saikkonen and Kaarakainen, 2021). Ample digital resource allocation enables teachers to use digital tools more frequently in their teaching, thereby strengthening their self-efficacy and contributing to the enhancement of digital literacy (Chao et al., 2016). Thus, self-efficacy serves as a critical mediating factor in the relationship between digital resource allocation and teachers’ digital literacy. When teachers exhibit higher levels of self-efficacy, they are more likely to demonstrate advanced digital literacy in their daily teaching practices (Awofala et al., 2019). This underscores the importance of fostering self-efficacy in efforts to improve teachers’ digital competencies and further supports the relevance of Bandura’s (2006) self-efficacy theory in the field of educational technology.

### 4.3 Mediating role of learning motivation between digital resource allocation and kindergarten teachers’ digital literacy

The study further discovered that learning motivation serves as a mediating factor between digital resource allocation and kindergarten teachers’ digital literacy, with an effect value of 4.15%. Structural equation model analysis revealed a significant positive correlation between digital resource allocation and learning motivation, consistent with previous research findings (Lin et al., 2017). A rich allocation of digital resources helps stimulate teachers’ learning motivation. When teachers exhibit stronger learning motivation, they are more likely to actively integrate digital technologies into their daily teaching practices, thereby enhancing their digital literacy. The finding that learning motivation mediates the relationship between digital resource allocation and digital literacy aligns closely with Gopalan et al.’s (2017) principles of academic motivation, which emphasize that motivational states are significantly shaped by external learning environments and perceived support. In the context of this study, kindergartens with abundant digital resources create an environment that signals institutional support and opportunity, thereby fostering teachers’ learning motivation to adopt and integrate digital tools in their teaching practices. This heightened motivation, in turn, contributes to improved levels of digital literacy. This supports Gopalan et al.’s assertion that motivational dynamics act as mediators between contextual factors and performance outcomes, underscoring the importance of not only infrastructure investment but also motivation-building strategies in digital literacy development.

### 4.4 Chain mediating role of self-efficacy and learning motivation between digital resource allocation and digital literacy of kindergarten teachers

This study further confirmed the chain mediating role of self-efficacy and learning motivation in the relationship between digital resource allocation and kindergarten teachers’ digital literacy. The findings indicate that digital resource allocation influences teachers’ digital literacy not only through the individual mediating effects of self-efficacy and learning motivation but also through a combined chain mediation path involving both, with a mediation effect value of 10.58%. Previous research suggests that teachers’ perception of support through digital resource allocation enhances their self-efficacy (Beck and Schmidt, 2015), and individuals with higher self-efficacy typically display stronger learning motivation (Bandura, 1977). Motivated by this learning drive, teachers often demonstrate higher levels of digital literacy in the application of digital technologies and teaching practices.

### 4.5 Research implications

This study incorporates self-efficacy and learning motivation theories into the research on kindergarten teachers’ digital literacy, confirming their mediating roles between digital resource allocation and teachers’ digital literacy. The findings highlight the complexity of the relationships among these variables. From a practical standpoint, the study reveals the key mechanisms influencing the development of digital literacy among kindergarten teachers, offering both empirical evidence and practical guidance for its enhancement. Firstly, at the policy level, the government should continue to strengthen support for educational digitalization, particularly in early childhood education, by improving digital infrastructure and providing kindergarten teachers with abundant, high-quality digital resources (Liu et al., 2024). Secondly, kindergarten teachers should focus on cultivating their self-efficacy and learning motivation (Pajares, 1997). Thirdly, given the complex nature of the chain mediating effect, improving digital resource allocation can offer essential external support, which in turn stimulates teachers’ self-efficacy and learning motivation, ultimately promoting the advancement of their digital literacy.

### 4.6 Limitations and future research

This study still has certain limitations. Firstly, the sample of 317 kindergarten teachers was drawn solely from the southeastern region of China, which limits the external validity and generalizability of the findings. Future research should include samples from a wider range of geographical areas, potentially expanding to a national level, to improve the representativeness and universality of the results. Secondly, the use of a cross-sectional design restricts the study to identifying correlational rather than causal relationships. Thus, future studies can adopt longitudinal tracking and experimental designs to manipulate and intervene in mediating variables, thereby providing a more rigorous test of the theoretical model. Thirdly, this study examined only the mediating roles of self-efficacy and learning motivation in the relationship between digital resource allocation and kindergarten teachers’ digital literacy. However, other potential mediating or moderating variables such as institutional digital culture, digital policy support, and teachers’ digital skills may exist. Future research should broaden the framework to explore the multifaceted mechanisms through which digital resource allocation influences teachers’ digital literacy. Additionally, reliance on self-reported questionnaires may introduce subjective bias, potentially causing discrepancies between reported data and actual conditions. Future studies should consider incorporating objective measurement tools, such as behavioral observations and performance assessments, to enhance data accuracy and the credibility of the findings. Finally, it should be noted that although the overall model fit was acceptable, the RMSEA value (0.088) slightly exceeded the conventional cutoff of 0.08. This minor deviation should be interpreted with caution, but the strong performance of other indices (CFI, TLI, SRMR) indicates the model remains robust.

## 5 Conclusion

Grounded in self-efficacy and learning motivation theories, this study investigates the associative mechanisms linking digital resource allocation to kindergarten teachers’ digital literacy. Employing structural equation modeling, the research examines the path relationships among digital resource allocation, self-efficacy, learning motivation, and digital literacy. The findings reveal that both self-efficacy and learning motivation independently mediate the relationship between digital resource allocation and digital literacy. Furthermore, self-efficacy and learning motivation jointly exhibit a chain mediating effect in this relationship. On the one hand, the study extends the application of self-efficacy and learning motivation theories to the domain of kindergarten teachers’ digital literacy, thereby enriching the theoretical landscape. On the other hand, by identifying key influencing factors, it offers practical insights into strategies for improving teachers’ digital competencies. Accordingly, it is recommended that governments increase investment in digital resources for kindergartens to provide teachers with abundant and accessible teaching tools. Simultaneously, kindergarten teachers should actively cultivate their self-efficacy and learning motivation, striving to acquire digital knowledge and skills to comprehensively enhance their digital literacy.

## Data Availability

The original contributions presented in the study are included in the article/Supplementary material, further inquiries can be directed to the corresponding author.
